# The Impact of Long COVID on Cognitive Functioning

**DOI:** 10.3390/jintelligence14080157

**Published:** 2026-08-01

**Authors:** Karen L. Siedlecki, Veronika Kobrinsky

**Affiliations:** 1 Department of Psychology, Fordham University, 113 W 60th St., New York, NY 10023, USA; 2 Department of Psychology, University of Wisconsin, 1202 W Johnson St., Madison, WI 53706, USA; kobrinsky@wisc.edu

**Keywords:** long COVID, objective cognition, subjective cognition, mental fatigue

## Abstract

This study systematically examined whether long COVID negatively impacts subjective and objective measures of cognition in a community-based sample of 251 participants. Participants completed a battery of neurocognitive tasks (including assessments of short-term memory, working memory, episodic memory, processing speed, executive functioning, and reasoning) and provided self-ratings of their cognition (mental fatigue and subjective cognitive difficulties), depressive symptoms, the impact of symptoms on their life, and completed surveys assessing other psychosocial outcomes. Subgroups included individuals with no known lifetime history of COVID-19 illness (*n* = 66), those who had recovered from a short-term COVID infection (*n* = 119), and those who had been diagnosed with long COVID (*n* = 62). Analyses indicated that long COVID was associated with worse performance on measures of processing speed and executive functioning. In addition, those with a history of long COVID reported higher levels of mental fatigue and subjective cognitive difficulties compared to the other subgroups. Analyses within the long COVID subsample indicated that after controlling for covariates, depressive symptoms were significantly associated with mental fatigue and self-reported cognitive difficulties, and ratings of symptom impact also significantly predicted mental fatigue. These findings reinforce the value of incorporating both subjective and objective assessments when evaluating cognitive outcomes in long COVID, as each captures distinct aspects of functioning. Furthermore, these findings underscore the importance of assessing subjective cognitive concerns and functional symptom burden in clinical encounters, even when objective performance is generally unimpaired.

## 1. The Impact of Long COVID on Cognitive Functioning

While the initial variant of COVID-19 was characterized primarily as a respiratory illness, subsequent research has demonstrated neurological involvement and measurable changes in brain structure among COVID-19 survivors (e.g., Thapaliya et al., 2025; Zhao et al., 2024). Neurological symptoms of COVID-19 include headaches, alterations in smell and taste, fluctuations in consciousness, confusion, and impairments in short-term memory, working memory, processing speed, executive functioning, and global cognition (e.g., Almeria et al., 2020; Delgado-Alonso et al., 2022; Tabacof et al., 2022; Zhou et al., 2020). A recent systematic review and meta-analysis conducted by Fanshawe et al. (2025) examined cognitive impairment following COVID-19 infection. They found that following a COVID-19 infection, individuals performed worse on measures of executive function, learning and memory, complex attention, language, and perceptual motor function compared to control participants. Fanshawe et al. suggest that these findings indicate that COVID-19 infection is associated with a more generalized disruption in brain function rather than being domain-specific.

“Long COVID” (or “long-haul COVID-19” or “Post-acute Sequelae of COVID-19”) refers to a condition in which individuals continue to experience symptoms of COVID-19 for an extended period of time post-infection. There is no consensus on how long symptoms need to be present to be categorized as long COVID. The Centers for Disease Control and Prevention in the U.S. specifies that symptoms should be present for at least four weeks, whereas the World Health Organization specifies that long COVID symptoms must be present for at least two months and evident within three months after acute infection. Although the precise mechanisms underlying long COVID remain an area of active investigation, current evidence points to several interconnected pathophysiological processes. These include viral persistence, wherein SARS-CoV-2 may form tissue reservoirs that evade immune clearance while sustaining chronic inflammatory responses; immune dysregulation, characterized by cytokine imbalances, T-cell exhaustion, and persistent upregulation of proinflammatory pathways such as IL-6 and JAK-STAT signaling; autoantibody production; endothelial dysfunction and micro-clotting, which may compromise cerebrovascular integrity; and mitochondrial dysfunction (Castanares-Zapatero et al., 2022; Aid et al., 2026; Gupta et al., 2025). Notably, neuroinflammation resulting from these mechanistic processes has been specifically implicated in the cognitive symptoms characteristic of long COVID, including brain fog and memory complaints (Castanares-Zapatero et al., 2022; Gupta et al., 2025). Long COVID has emerged as a public health concern, with growing research indicating that people who experienced acute COVID-19 infections often continue to face challenges—even after they have fully recovered from the acute infection stage. Estimates of the prevalence of long COVID are variable because there is not one way to operationalize the condition. However, in 2023, the CDC reported 6.0% of U.S. adults reported experiencing long COVID (Ford et al., 2023).

Individuals with long COVID experience residual symptoms and ongoing health challenges for weeks or months post-infection. A meta-analysis of symptoms prevalent in long COVID showed that a substantial percentage of individuals experience, among other symptoms, persistent brain fog, memory complaints, and attentional complaints (Premraj et al., 2022). For example, Graham et al. (2021) examined a non-hospitalized sample of COVID-19-positive and COVID-19-negative individuals with neurologic symptoms lasting longer than six weeks. They found that more than half of individuals with long COVID-19 performed below average on measures of working memory and attention. These participants also reported persistent brain fog and fatigue that negatively affected their quality of life.

Earlier work in our lab (Kobrinsky et al., 2024) found preliminary evidence that long COVID was associated with worse subjective assessments of cognition, but not worse performance on objective measures of cognition. This is not surprising considering that subjective assessments and performance-based measures are likely measuring distinct dimensions of individual differences (e.g., Hertzog et al., 2018). Whereas subjective assessments comprise one’s perception of their cognitive abilities, objective assessments comprise performance on neurocognitive tasks. Subjective and objective measures are generally only weakly related. A meta-analysis examining the relationship between subjective cognitive complaints and general measures of cognition found that the relationship among these variables is significant but weak and, notably, subjective cognitive complaints may be more strongly associated with depression than objective measures of cognition (Burmester et al., 2016). Rennison et al. (2024) examined subjective and objective cognition in a sample of individuals with long COVID. These individuals reported impairments in daily life as assessed by the British Columbia Post Concussion Symptom Inventory, including poor concentration, memory problems, and difficulty reading. However, mean performance on a battery of neurocognitive tasks did not yield any objective deficits, defined as performance at or below the 8th percentile. Those participants who were categorized as having a high psychological symptom burden reported more subjective cognitive difficulties than those with low psychological symptom burden, but no differences were evident on the objective cognitive assessments. Others have found a significant relationship between subjective cognition (measured with the Perceived Deficits Scale) and objective measures of cognition in a sample of individuals with long COVID (Kwan et al., 2024). However, consistent with previous work, these effects were generally small.

Despite growing evidence that long COVID affects cognitive functioning, studies vary considerably in their inclusion of both subjective and objective cognitive assessments, and few have simultaneously examined predictors of both cognitive domains within long COVID samples. Therefore, the primary goal of the current study is to examine the ways in which long COVID impacts cognitive functioning. Using a design similar to our current study, Bland et al. (2024) examined both subjective and objective cognitive function in 162 individuals: 50 of whom had long COVID, 59 who had COVID but had recovered, and 53 who never tested positive for COVID. They found a main effect of COVID group such that long COVID participants reported worse subjective cognition, but this effect was eliminated when stress and fatigue were included in the model. Furthermore, Bland et al. (2024) found that subjective assessments and objective assessments of cognition were not significantly related to one another.

Because objective assessments are not expected to account for a substantial amount of variance in subjective assessments of cognition, another goal of the current study is to examine potential predictors of subjective assessments of cognition. Research has consistently demonstrated a robust relationship between subjective assessments of cognition and depressive symptoms (e.g., Zlatar et al., 2018), and Bland et al. (2024) reported an association between subjective cognition and stress and fatigue. A recent systematic review and meta-synthesis examining predictors of subjective cognitive dysfunction in patients with nondementia-related chronic illnesses (including long COVID) found that illness symptoms were linked to subjective assessments of cognitive dysfunction (Cuevas et al., 2024). Other research have demonstrated that aspects of well-being may be associated with cognition, including hopefulness (Lee et al., 2025). Hopefulness, a modifiable positive emotional state, may be protective against negative self-assessments of cognition. Understanding protective factors that influence cognitive outcomes is important for developing specific, person-centered interventions for individuals experiencing long COVID symptoms.

In summary, the main purpose of the current study was to further examine whether long COVID negatively impacts subjective and objective measures of cognition. Additional goals include a) examining the relationship between the subjective and objective measures, and b) examining which variables within the long COVID subsample were predictive of subjective cognition, including depressive symptoms, stress, hopefulness, and the functional impact of symptoms.

## 2. Methods and Materials

### Participants and Procedures

Participants comprised 251 community-based adults with a mean age of 43.80 years (*SD* = 17.01, range = 18–79). Individuals were classified into one of three groups based on self-reported COVID-19 history. Those who reported no known lifetime history of COVID-19 infection were assigned to the “no COVID” group. Those who reported a prior COVID-19 infection from which they had fully recovered were assigned to the “recovered COVID” group. Those who reported having been diagnosed with long COVID by a healthcare provider were assigned to the “long COVID” group. Classifications were not verified through clinical records or medical reports. Participants were recruited through regional healthcare clinics, the COVID-19 Longhauler Advocacy Project (C19LAP, n.d.), and ResearchMatch.org, which is a “national health volunteer registry that was created by several academic institutions and supported by the U.S. National Institutes of Health as part of the Clinical Translational Science Award (CTSA) program. ResearchMatch has a large population of volunteers who have consented to be contacted by researchers about health studies for which they may be eligible” (ResearchMatch, 2022). Inclusion criteria included being 18 years of age or older, fluent in spoken and written English, United States residence, and correctly answering attention confirmation items. Three attention confirmation items were embedded into the survey. These items asked participants to select a specific response (e.g., “For psychometric purposes, please select ‘Agree’ for this item”). Nine (3.46%) of the original 260 participants responded incorrectly to more than one attention confirmation item and were thus excluded from the current analyses, resulting in an analytic sample of 251.

The study procedure consisted of an online screening and a detailed informed consent survey, which provided information including details about study tasks, the voluntary nature of the study, benefits, risks, and expected time commitment. Participants who provided consent were scheduled for a Zoom research visit in which trained research assistants administered cognitive tasks and provided a link to an online survey. Data were collected between April 2023 and June 2024. Following completion of the study, participants were provided with a $15 electronic Amazon gift card and a list of COVID-19 and mental health resources. Participant consent was provided online prior to the study session, and verbal consent was obtained prior to starting the study session for each participant.

## 3. Measures

### 3.1. Objective Cognitive Functioning

Five verbally adapted tasks and one visually presented task were administered to assess multiple cognitive domains. Short-term and working memory were examined with the Forward and Backward Digit Span task wherein an examiner read aloud a series of digits that increased in length after each successful retrieval, and participants were asked to repeat the sequence in the same (forward condition) or reverse (backward condition) order of that presented (e.g., Wechsler, 1997a). The task was discontinued after errors on two consecutive trials or a maximum of 12 digits recalled. Higher values on the digit span are reflective of better short-term memory performance (forward digit span) and working memory (backward digit span). Processing speed and executive functioning were measured with an oral version of the Trail Making Test (TMT; Partington & Leiter, 1949). Part A entailed counting aloud from one to 25 and reciting the alphabet as quickly as possible. Part B consisted of two conditions where participants were instructed to alternate between recalling ascending numbers and letters, beginning with “1” in Condition 1 and “A” in Condition 2. Part B was discontinued at “13” or “M”, depending on the condition. Higher values indicate slower speed, reflective of slower processing speed and worse executive functioning performance. An Immediate Recall Test (e.g., Wechsler, 1997b) was used to measure episodic memory. In this task, participants were instructed to verbally recall as many of the 12 words that were read to them by the interviewer. The score represents the number of correctly recalled words after four separate trials, with higher scores indicating better performance. Finally, reasoning/fluid ability was assessed with a matrix reasoning task (e.g., Raven et al., 1998), in which participants were allotted 10 min to complete a series of pattern completion tasks. Higher scores indicate better performance.

### 3.2. Subjective Cognitive Functioning

Participants reported changes in perceived cognitive functioning and the presence of several symptoms (e.g., forgetfulness, difficulty recalling things) during the COVID-19 pandemic (De Pue et al., 2021) on a 5-point Likert scale (1 = A lot less than before to 5 = A lot more than before). The total score (labeled subjective cognitive difficulties) was calculated by summing individual items, with higher numbers reflecting greater cognitive difficulties. The variable of mental fatigue was measured with the 9-item Brief Mental Fatigue Questionnaire (Bentall et al., 1993), which asked participants about their current cognitive health compared to their perceptions of pre-pandemic functioning. Cognitive experiences (e.g., spells of confusion, poor concentration) were rated on a Likert scale from 1 = Not at all to 5 = Very much. The total score comprised an average of the items and higher scores correspond to greater mental fatigue.

### 3.3. Qualtrics Questionnaire

The full Qualtrics survey is provided in Supplementary Materials.

Demographics. Participants were asked to provide non-identifying information about their age, education level, gender, and racial/ethnic identities.

COVID-19 Characteristics. Multiple components of COVID-19, including past and current symptoms, illness duration, medical comorbidities, viral complications, treatment, and hospitalization history, were examined using a standard measure adapted from a primary care clinic and used in previous research (Kobrinsky et al., 2024). Long COVID status was determined by asking participants whether they had been diagnosed with long COVID. Participants were also asked to provide the approximate date of their first infection. This date was used in conjunction with the survey completion date to calculate the time elapsed between first infection and study participation for each participant in the long COVID subsample.

Depressive Symptoms. The past two-week frequency of depressed mood and anhedonia, the state of diminished interest or pleasure in engaging in previously enjoyable activities, was examined with the 2-item Patient Health Questionnaire-2 (PHQ-2; Kroenke et al., 2003). Participants were asked to rate how often they have been bothered by the following two symptoms of depression: (1) Little interest or pleasure in doing things and (2) Feeling down, depressed, or hopeless—on a 4-point scale ranging from 0 (*Not at all*) to 4 (*Nearly every day*). The total score was derived from a sum of both items, with higher scores indicating greater depressive symptoms.

Hopefulness. Participants were asked to indicate their level of agreement with the statement “I am hopeful about the future following COVID-19” on a 5-point scale ranging from 0 (*Strongly disagree*) to 5 (*Strongly agree)* to gauge their level of hopefulness, which is an item used in previous research (Hicks & McFarland, 2020). Higher scores represent more hopefulness following the COVID-19 pandemic.

Symptom Impact on Life. To examine the impact of COVID-19 symptoms on quality of life, participants were asked to think about “How much have these symptoms negatively impacted your quality of life” with responses ranging from 0 (*Not at all*) to 10 (*Very much so, affecting all or nearly all aspects of your life*). Higher numbers indicate a greater negative impact of symptoms on perceived quality of life. This item was developed by the research team for the purposes of the current study.

Perceived Stress Scale Modified for COVID-19. (PSS; Eubank et al., 2021). A modified version of the PSS was administered to assess stress associated with the COVID-19 pandemic. Participants responded to 13 items (such as “Felt that you were unable to control the important things in your life” and “Felt nervous and stressed”) regarding the pandemic. They responded to how often they experienced each item on a scale of 1 (*never)* to 5 (*very often*). A mean score was calculated after reverse-scoring relevant items. Higher values indicate higher levels of stress. In the current sample, alpha = .92 for the 13 items.

## 4. Data Analytic Plan

Analyses were conducted using SPSS 29.0 and listwise deletion was used to handle missing data. Due to underpowered subsample sizes, the analyses including gender identity included only those identifying as female or male (*n* = 239; 95.22% of the entire sample). To examine differences among groups, Multivariate Analysis of Covariance (MANCOVA) models were conducted with age, race/ethnicity, gender, and depressive symptoms as covariates, which are variables shown to be associated with subjective and objective measures of cognition in previous research. Pairwise comparisons were performed to identify which groups statistically differed from each other.

To examine the relationship between the subjective and objective measures zero-order correlations and partial correlations were conducted.

To examine predictors of subjective cognition within the long COVID subsample, zero-order correlations were examined first. Next, hierarchical regression models were conducted to determine which variables were unique predictors of the subjective cognitive assessments.

An alpha of 0.05 was used to determine significance.

## 5. Results

Of the entire sample, 66 individuals (26.30%) had no known lifetime history of COVID-19 illness (“no COVID” group), 119 (47.41%) reported short-term infection (“recovered COVID” group), and 62 (24.70%) indicated that they had been diagnosed with long COVID (“long COVID” group). Participants were a majority White (74.1%), followed by Black (14.7%), Asian/Asian American (9.2%), Hispanic/Latinx (8.0%), American Indian/Alaskan Native (3.6%) and 0.08% were self-described. Participants could select all the categories that applied so the total percentage exceeds 100%. The race/ethnicity variable was dichotomized into two racial/ethnic categories, White (*n* = 169, 67.3%) and Person of Color (POC; *n* = 82, 32.7%), to account for underpowered subsamples and participants endorsing multiple categories. Additional demographic and COVID-19 characteristics are presented in Table 1.

### 5.1. Differences Among COVID Groups

To examine differences among the COVID sub-groups, MANCOVAs were conducted with age, education, race/ethnicity, gender, and depressive symptoms included as covariates in each analysis.

First, the six objective cognition variables were examined in a MANCOVA. There was a statistically significant difference between the COVID groups on the combined dependent variables after controlling for age, education, race, gender, and depressive symptoms, F(12, 426) = 1.829, *p* < .05, Wilks’ Λ = 0.904, partial η^2^ = 0.049. Pairwise comparisons with Bonferroni correction indicate that those with long COVID performed worse than those with recovered COVID on Trails A, and those with long COVID performed worse on Trails B compared to both the recovered and no COVID groups (see Table 2).

Next, the two subjective cognition variables were included as dependent variables in a MANCOVA. There was a statistically significant difference between the COVID groups on the combined dependent variables after controlling for age, education, race, gender, and depressive symptoms, *F*(4, 444) = 16.572, *p* < .001, Wilks’ Λ = 0.757, partial η^2^ = 0.130 (see Table 3). Pairwise comparisons with Bonferroni correction indicate that those with long COVID reported higher levels of subjective cognitive difficulties and mental fatigue as compared to both groups.

### 5.2. Zero-Order Correlations

Analyses were then conducted to examine zero-order correlations among the study variables (see Table 4) across the entire sample to evaluate the relationships among subjective and objective cognitive functioning. Notably, mental fatigue was weakly but significantly correlated (*r*s range from −0.15 to 0.28) with performance on most of the objective assessments of cognition including digit span forward, digit span backward, word recall, Trails A, and Trails B such that higher levels of mental fatigue were associated with worse cognitive performance. Subjective cognitive difficulties were also significantly weakly correlated with performance on word recall (*r* = −0.14, *p* < .05) and Trails B (*r* = 0.26, *p* < .01). Both subjective cognitive difficulties (*r* = 0.38, *p* < .01) and mental fatigue (*r* = 0.57, *p* < .01) variables were significantly associated with depressive symptoms.

Results from partial correlations controlling for depressive symptoms showed that mental fatigue remained significantly correlated with the same objective assessments of cognition, except digit span backward (*r* = −0.13, *p* = .05). After statistically accounting for the influence of depressive symptoms, subjective cognitive difficulties were no longer significantly associated with word recall (*r* = −0.12, *p* = .058) but remained significantly associated with Trails B (*r* = 0.21, *p* < .01). The correlation (*r* = 0.16, *p* < .05) between subjective cognitive difficulties and matrix reasoning became significant once depressive symptoms were controlled for.

### 5.3. Long COVID Specific Analyses

Analyses were conducted to examine predictors of the outcomes within the long COVID subsample (*n* = 62). Of those who reported being diagnosed with long COVID, 49 (79%) reported they were still experiencing symptoms. Half of the participants (*n* = 31) reported that they had been experiencing symptoms for greater than six months; 29% reported the symptoms lasted between 0–4 weeks, 9.7% reported symptoms lasting 1–2 months, and 8.1% reported they had experienced symptoms for 2–5 months. Participants were asked whether they required hospitalization for their COVID-19 infection. Twelve (19.4%) required hospitalization and 50 (80.6%) did not require hospitalization. Hospitalization was not significantly associated with any of the outcome variables among the participants diagnosed with long COVID (see Table 5).

Time since first COVID-19 infection was calculated for all 62 participants in the long COVID subsample. Time since infection ranged from 2.9 to 51.6 months (*M* = 27.6, *SD* = 12.7), indicating that participants were, on average, 2.3 years post first infection at the time of the study. Zero-order correlations indicated that time since infection was not significantly associated with either subjective cognitive outcomes (subjective cognitive difficulties: *r* = −0.12, *p* = .34; mental fatigue: r = −0.01, *p* = .97) or with most objective cognitive outcomes (forward digit span: *r* = −0.12, *p* = .35; backward digit span: *r* = −0.02, *p* = .90; word recall: *r* = −0.10, *p* = .45; Trails B: *r* = −0.04, *p* = .75; matrix reasoning: *r* = −0.13, *p* = .32). A significant positive association emerged between time since infection and Trails A (*r* = 0.38, *p* = .003), such that longer time since infection was associated with slower processing speed. This association remained significant after controlling for age, education, gender, and race/ethnicity (*p* = .02).

Zero-order correlations in the long COVID subsample (see Table 5) indicate that ratings of symptoms on quality of life were significantly associated with subjective cognitive difficulties (*r* = 0.44, *p* < .01) and mental fatigue (*r* = 0.47, *p* < .01). Mental fatigue was also significantly positively associated with stress (*r* = 0.37, *p* < .01) and depressive symptoms *(r* = −0.31, *p* < .05), and significantly negatively associated with hopefulness (*r* = −0.26, *p* < .01). Subjective cognitive difficulties were significantly associated with age (*r* = 0.33, *p* < .01) and race/ethnicity such that white individuals reported higher levels of difficulties as compared to POC.

Subsequent separate hierarchical regression analyses were conducted for each subjective cognitive variable. Age, race/ethnicity, gender, and education were included in block 1 of the analysis for both dependent variables.

When mental fatigue was examined as the dependent variable, symptom impact, stress, hopefulness, and depressive symptoms were included in block 2 since those variables exhibited significant zero-order correlations with mental fatigue. Results of the hierarchical regression indicate that after controlling for demographic variables, symptom impact (*β* = 0.36, *p* < .05) and depressive symptoms (*β* = 0.31, *p* < .05) remained significant predictors of mental fatigue (see Table 6).

When subjective cognitive difficulties were examined as the dependent variable, block 2 included just depressive symptoms since it was the only variable that had a significant association with subjective cognitive difficulties. After statistically controlling for demographic variables, depressive symptoms (*β* = 0.35, *p* < .05) remained a significant predictor of subjective cognitive difficulties.

## 6. Discussion

The first goal of the current study was to examine the influence of long COVID on both subjective and objective assessments of cognition. Consistent with our hypotheses, individuals with long COVID performed worse than those with recovered COVID on a measure of processing speed (Trails A), and performed worse than both the recovered and no COVID groups on a measure of executive functioning (Trails B). These findings align with a growing body of literature documenting executive functioning impairments in long COVID (Dacosta-Aguayo et al., 2024; Hadad et al., 2022; Hampshire et al., 2024; Herrera et al., 2023; Kozik et al., 2023; Krishnan et al., 2022). For instance, in their review of cognitive sequelae of long COVID, Nicotra et al. (2023) reported that attention and executive functions were the most commonly impacted cognitive domains; they found that attention/executive function domains were adversely affected in 90% cases that demonstrated an impairment in objective cognitive assessments. Executive functioning encompasses processes such as inhibition, cognitive flexibility, and working memory, capacities that underlie complex daily behaviors, including multitasking, planning, and health self-management. As such, even modest impairments in these domains may carry meaningful consequences for daily functioning, academic or occupational performance, and health behaviors (Foster & Hershey, 2011; Williams et al., 2017). Taken together, our findings underscore the importance of targeting executive functioning specifically in both clinical evaluations and research investigations of long COVID, rather than relying solely on global cognitive screening measures. An additional goal of the study was to examine the relationship between subjective and objective assessments of cognition. In this sample, higher levels of mental fatigue were associated with poorer performance across most objective cognitive tasks, suggesting that mental fatigue may play a meaningful role in day-to-day cognitive efficiency. Subjective cognitive difficulties also showed small but significant associations with performance on word recall and Trails B, indicating that individuals’ perceptions of their cognitive challenges are not entirely disconnected from objective performance. Consistent with previous work, these associations were generally small, suggesting that subjective and objective measures assess distinct aspects of cognition. Also consistent with previous work, both subjective assessments of cognition had moderate-to-strong significant associations with depressive symptoms. Importantly, partial correlations indicated that the relationships between subjective and objective cognition persisted even after accounting for depressive symptoms, suggesting that these associations cannot be entirely explained by depressive symptoms. Taken together, these findings provide evidence that while subjective cognition and objective cognition represent distinct dimensions of individual differences, they share some common variance.

The final goal of the study was to identify which variables uniquely predicted subjective assessments of cognition within the subsample of participants with long COVID. Brain fog, memory and attentional complaints, and difficulties concentrating are widely reported hallmarks of long COVID and are often described as among the most disruptive symptoms. These cognitive concerns can interfere with daily functioning, work productivity, and overall quality of life, making it important to understand the factors that shape individuals’ perceptions of their cognitive abilities. Examining predictors of subjective cognition may help clarify why some individuals experience noticeable cognitive difficulties despite relatively modest objective impairments, and may provide insight into the mechanisms through which long COVID is associated with day-to-day cognitive experiences. Our results show that after statistically controlling for demographic variables, depressive symptoms accounted for unique variance in both mental fatigue and subjective cognitive difficulties (i.e., self-reported perceptions of cognitive functioning) in individuals with long COVID. This finding suggests that affective processes may contribute to how individuals appraise their cognitive capacity in the context of long COVID. These associations suggest that depression-related mechanisms, such as reduced motivation, attentional disruption, or negative cognitive bias (Kircanski et al., 2012), may partially underlie self-reported cognition, independent of demographic factors. This also suggests that interventions targeting mood symptoms may be essential for improving subjective cognition.

In addition, functional symptom impact was a significant unique predictor of mental fatigue. This result indicates that mental fatigue is not only associated with affective factors but may also be associated with the strain of managing ongoing symptoms. Higher symptom impact may negatively impact cognitive resources through mechanisms such as increased self-monitoring, disrupted sleep, or heightened physiological stress.

We also examined whether the time elapsed since the first COVID-19 infection was associated with cognitive outcomes within the long COVID subsample, given that prior research has documented both persistent symptoms and substantial spontaneous recovery over time (Delgado-Alonso et al., 2022; Fanshawe et al., 2025). With the exception of Trails A, time since infection was not significantly associated with subjective or objective cognitive measures, suggesting that the duration of illness since initial infection did not broadly shape cognitive functioning in this sample. The significant association between time since infection and Trails A may reflect the possibility that individuals infected earlier in the pandemic experienced more severe acute illness or had less access to emerging treatments, such that longer time since infection indexes illness severity rather than duration per se. Future longitudinal studies with larger samples and more granular illness severity data will be needed to disentangle these findings.

## 7. Limitations and Future Directions

The present study is among the first to use both subjective and objective cognitive assessments, which allowed for a more comprehensive evaluation of cognitive functioning than studies relying on a single measurement approach. Additionally, the inclusion of three comparison groups (no COVID, recovered COVID, and long COVID) allowed for a more nuanced interpretation of group differences in cognitive outcomes than a simple case–control design. The community-based recruitment strategy further enhanced ecological validity relative to studies drawing exclusively from hospitalized or clinical samples. Finally, the examination of predictors of subjective cognition within the long COVID subsample addresses a relatively unexplored question in the literature and has direct clinical implications.

Nonetheless, the aforementioned strengths should be considered alongside several limitations that point to important directions for future research. Our study sample comprised a majority of White women; the results may therefore lack generalizability to more diverse samples. Relatedly, because participation required computer and internet access and recruitment occurred through ResearchMatch.org and social media, the study was accessible only to individuals with these resources. As a result, the sample may differ systematically from those without such access, introducing potential selection bias and further limiting the generalizability of our findings. Future studies should endeavor to recruit more diverse samples, especially considering that the COVID-19 pandemic has highlighted the inequities that exist in the American healthcare system (e.g., Lopez et al., 2021). Another limitation was that long COVID status was ascertained by self-report (and not verified through clinical records). We also did not assess whether the participant was currently experiencing symptoms of long COVID or symptoms of an acute infection. In addition, we did not collect data regarding the exact number of weeks participants had experienced symptoms but rather had participants select pre-determined categories, which limited our ability to filter participants based on symptom duration.

Data collection took place online in order to allow flexibility in scheduling, reduce the burden on participants, and to remove transportation barriers. However, the online administration of the study necessitated that Trail Making Task (TMT) be administered auditorily (rather than using pen and paper, which is the traditional way that the TMT is administered). While there is evidence that the auditory version demonstrates adequate reliability and validity (Mrazik et al., 2010), administering an auditory version of the TMT may have decreased the validity of the assessment.

## 8. Conclusions

The current study replicated previous work indicating that long COVID was associated with worse subjective assessments of cognitive functioning (Graham et al., 2021; Premraj et al., 2022), as compared to groups with recovered COVID and no COVID. Long COVID was also associated with worse performance on measures of processing speed (compared to the recovered COVID group) and executive functioning (compared to both groups). Results of this study also provided evidence that subjective assessment of cognition was correlated with objective assessments, even after accounting for the influence of depressive symptoms, but these effects were generally small, consistent with previous research (Burmester et al., 2016). Finally, within the long COVID subsample, depressive symptoms were a unique predictor of mental fatigue and cognitive difficulties, even after controlling for demographic variables. Mental fatigue was also significantly predicted by symptom impact. These results reinforce the value of incorporating both subjective and objective assessments when evaluating cognitive outcomes in long COVID, as each captures distinct aspects of functioning. Furthermore, these findings underscore the importance of assessing subjective cognitive concerns and symptom burden in clinical encounters, even when objective performance is generally unimpaired.

## Figures and Tables

**Table 1:** Participant Demographics and COVID-19 Characteristics (*N* = 251).

	Total Sample	No COVID	Recovered COVID	Long COVID
	(*N* = 251)	(*n* = 66)	(*n* = 123)	(*n* = 62)
Characteristic	*M* (*SD*) or *N* (%)	*M* (*SD*) or *N* (%)	*M* (*SD*) or *N* (%)	*M* (*SD*) or *N* (%)
Age	43.80 (17.01)	48.75 (18.65)	41.60 (16.78)	42.86 (14.66)
Education	17.09 (2.89)	17.35 (3.22)	17.22 (2.59)	16.54 (3.040)
Gender				
Male	62 (24.7)	27 (40.9)	24 (19.5)	11 (17.70)
Female	177 (70.5)	39 (59.1)	95 (77.2)	43 (69.40)
Transgender female	1 (0.4)	0 (0.0)	0 (0.0)	1 (1.60)
Gender queer	2 (0.8)	0 (0.0)	0 (0.0)	2 (3.20)
Non-binary	7 (2.8)	0 (0.0)	3 (2.4)	4 (6.50)
Self-described	2 (0.8)	0 (0.0)	1 (0.8)	1 (1.60)
Race/ethnicity				
White	186 (74.1)	42 (63.6)	93 (75.6)	51 (82.30)
Black or African American	37 (14.7)	10 (15.2)	20 (16.3)	7 (11.30)
Asian/Asian American	23 (9.2)	13 (19.7)	8 (6.5)	2 (3.2)
Hispanic/Latinx	20 (8.0)	2 (3.0)	14 (11.4)	4 (6.5)
American Indian/Alaskan Native	9 (3.6)	1 (1.5)	7 (5.7)	1 (1.6)
Self-described	2 (0.8)	1 (1.5)	1 (0.8)	0 (0.0)
Number of COVID-19 infections				
0	66 (26.3)	--	0 (0.0)	0 (0.0)
1	113 (45.0)	--	83 (67.5)	30 (48.4)
2	57 (22.7)	--	33 (26.8)	24 (38.7)
3	10 (4.0)	--	5 (4.1)	5 (8.1)
4	2 (0.8)	--	0 (0.0)	2 (3.2)
5 or more	3 (1.2)	--	2 (1.6)	1 (1.6)
COVID-19 treatment history				
Prescription medication(s)	65 (35.30)	--	30 (24.4)	35 (56.5)
Hospitalization	16 (8.70)	--	4 (3.3)	12 (19.4)
Self-reported COVID-19 symptom duration				
0–4 weeks	119 (47.4)	--	101 (82.1)	18 (29.0)
1–2 months	12 (4.8)	--	6 (4.9)	6 (9.7)
2–5 months	8 (3.2)	--	3 (2.4)	5 (8.1)
6 months or greater	35 (13.9)	--	4 (3.3)	31 (50.0)

**Table 2:** MANCOVA Results for Objective Cognition Dependent Variables Across COVID Groups.

	No COVID		Recovered COVID		Long COVID		Bonferroni Corrected *p*-Values		
Outcome	*M*_adj_ (SE)	95% CI	*M*_adj_ (SE)	95% CI	*M*_adj_ (SE)	95% CI	*p* ^1^	*p* ^2^	*p* ^3^
Matrix reasoning	7.37 (0.38)	6.63, 8.11	7.74 (0.28)	7.20, 8.29	7.50 (0.42)	6.67, 8.33	1.00	1.00	1.00
Digit span forward	6.74 (0.19)	6.37, 7.11	6.48 (0.14)	6.21, 6.75	6.42 (0.21)	6.00, 6.83	0.83	0.80	1.00
Digit span backward	5.16 (0.19)	4.78, 5.54	5.04 (0.14)	4.76, 5.32	4.92 (0.22)	4.49, 5.35	1.00	1.00	1.00
Word recall	33.91 (0.70)	32.52, 35.30	33.41 (0.52)	32.38, 34.43	32.83 (0.79)	31.27, 34.39	1.00	0.96	1.00
Trails A	7.81 (0.30)	7.21, 8.40	7.05 (0.22)	6.61, 7.48	8.03 (0.34)	7.36, 8.70	0.14	1.00	0.05
Trails B	25.64 (0.76)	23.60, 27.68	24.97 (0.76)	23.47, 26.48	29.85 (1.16)	27.56, 32.13	1.00	0.03	0.00

Note. Covariates included age, education, gender, race/ethnicity, and depressive symptoms. *p* ^1^ = *p* value for comparison between the No COVID and Recovered COVID groups; *p* ^2^ = *p* value for the comparison between the No COVID and Long COVID groups; *p* ^3^ = *p* value for comparison between the Recovered COVID and Long COVID groups.

**Table 3:** MANCOVA Results for Subjective Cognition Dependent Variables Across COVID Groups.

	No COVID		Recovered COVID		Long COVID		Bonferroni Corrected *p*-Values		
Outcome	*M*_adj_ (SE)	95% CI	*M*_adj_ (SE)	95% CI	*M*_adj_ (SE)	95% CI	*p* ^1^	*p* ^2^	*p* ^3^
Mental fatigue	1.98 (0.11)	1.77, 2.20	2.16 (0.08)	2.00, 2.32	3.08 (0.12)	2.85, 3.32	0.60	<.001	<.001
Subjective cognitive difficulties	3.29 (0.08)	3.13, 3.45	3.54 (0.06)	3.42, 3.66	4.25 (0.09)	4.07, 4.43	0.06	<.001	<.001

Note. Covariates included age, education, gender, race/ethnicity, and depressive symptoms. *p* ^1^ = *p* value for comparison between the No COVID and Recovered COVID groups; *p* ^2^ = *p* value for the comparison between the No COVID and Long COVID groups; *p* ^3^ = *p* value for comparison between the Recovered COVID and Long COVID groups.

**Table 4:** Correlations among Study Variables (Valid *N*s range from 243–251).

	1.	2.	3.	4.	5.	6.	7.	8.	9.	10.	11.	12.
1. Age	--											
2. Education	.13 *	--										
3. Gender (1 = male, 2 = female)	−.13	−.00	--									
4. Race/ethnicity (1 = White, 2 = POC)	−.29 **	−.13 *	−.04	--								
5. Depressive symptoms	−.19 **	−.23 **	.00	.05	--							
6. Matrix reasoning	−.28 **	.02	.18 **	−.15 *	−.06	--						
7. Digit span forward	−.10	.09	−.06	−.01	−.07	.19 **	--					
8. Digit span backward	−.06	.17 **	−.07	−.07	−.14 *	.19 **	.56 **	--				
9. Word recall	−.33 **	.15 *	.23 **	−.04	−.10	.26 **	.20 **	.35 **	--			
10. Trails A	.06	−.06	−.24 **	.12	.15 *	−.29 **	−.22 **	−.19 **	−.21 **	--		
11. Trails B	−.05	−.05	.08	.08	.19 **	−.20 **	−.44 **	−.43 **	−.14 *	.34 **	--	
12. Subjective cognitive difficulties	.06	−.07	.15 *	−.16 *	.38 **	.11	−.07	−.09	−.14 *	.07	.26 **	--
13. Mental fatigue	−.11	−.18 **	.11	.05	.57 **	−.07	−.15 *	−.17 **	−.20 **	.21 **	.28 **	.76 **

Note. POC = Persons of Color; * *p* < .05, ** *p* < .01.

**Table 5:** Correlations among Study Variables within the Long COVID subsample (Valid *N*s range from 52–61).

	1.	2.	3.	4.	5.	6.	7.	8.	9.	10.	11.	12.	13.	14.	15.	16.
1. Mental fatigue	--															
2. Subjective cognitive difficulties	.76 **	--														
3. Matrix reasoning	−.03	.05	--													
4. Digit span forward	−.24	−.23	.11	--												
5. Digit span backward	−.14	−.12	−.12	.62 **	--											
6. Word recall	−.24	−.16	.19	.23	.26 *	--										
7. Trails A	.14	.02	−.29 *	−.18	−.11	−.20	--									
8. Trails B	.11	.12	−.11	−.47 **	−.46 **	−.01	.39 **	--								
9. Symptom impact	.47 **	.44 **	.02	−.16	−.04	−.06	.12	−.13	--							
10. Stress	.37 **	.14	−.08	−.16	−.04	.19	−.10	−.13	.12	--						
11. Hopefulness	−.26 *	−.21	−.09	−.00	.25 *	.06	−.08	−.03	−.05	−.35 **	--					
12. Age	.22	.33 **	−.33 **	−.32 *	−.05	−.44 **	.14	−.02	.05	−.13	.07	--				
13. Education	.06	.19	.02	−.02	.06	.15	−.08	.06	.06	−.00	.21	.13	--			
14. Race/ethnicity (1 = White, 2 = POC)	−.19	−.45 **	−.26 *	.03	−.03	−.02	.00	−.07	−.32 *	.13	.10	−.17	−.17	--		
15. Gender (1 = male, 2 = female)	.16	.06	.35 **	−.13	−.27	.14	−.27	.10	.01	.26	−.24	−.22	−.09	.09	--	
16. Hospitalization (1 = yes, 2 = no)	.20	.21	−.08	.06	.18	.16	−.04	−.05	.04	.06	.15	−.11	.17	.07	.09	
17. Depressive symptoms	.31 *	.17	−.11	−.08	−.12	.00	.15	.09	.24	.27 *	−.32 *	−.10	−.36 **	.09	−.03	.17

*Note*. POC = Persons of Color; * *p* < .05, ** *p* < .01.

**Table 6:** Standardized Coefficients and Change Statistics from Block 2 in the Hierarchical Regression Models Predicting Subjective Cognition.

	Dependent Variable	
	Mental Fatigue	Subjective Cognitive Difficulties
Block 1 predictors		
Age	.39 **	.30 *
Gender (1 = male, 2 = female)	.07	.04
Education	.14	.09
Race/ethnicity (1 = White, 2 = POC)	.05	−.12
Block 2 predictor(s)		
Symptom impact	.36 *	-
Stress	.08	-
Hopefulness	.12	-
Depressive symptoms	.31 *	.35 *
Change statistics for Block 2		
R^2^ change	.25	.11
F change	4.02	5.83
*df*1	4	1
*df*2	34	37
*p*	.009	.021

*Note*. POC = Persons of Color; * *p* < .05, ** *p* < .01.

## Data Availability

The original contributions presented in this study are included in the article. Further inquiries can be directed to the corresponding author.

## Supplementary Materials

The following supporting information can be downloaded at: https://www.mdpi.com/article/10.3390/jintelligence14080157/s1.
