# Supplementary material for: The Impact of Long COVID on Cognitive Functioning
Source: J Intell. 2026 Aug 1;14(8):157. doi: 10.3390/jintelligence14080157 (PMC13514807; doi:10.3390/jintelligence14080157)
Supplement: Supplementary file 1 [file jintelligence-14-00157-s001.zip › jintelligence-4246209-supplementary.pdf]

CCP v.2

Start of Block: Mode of Completion

Q40
Welcome to Part 2 of the study!

We would now like you to complete this set of questionnaires. We expect Part 2 to take 15-20 minutes to complete.

As you progress through the survey, feel free to ask your examiner any questions you may have.

Before you begin, please enter your participant number provided to you by the examiner:

____

Page Break

Q1 Which type of device are you completing this survey on?

- ○ Computer (laptop) (1)
- ○ Computer (desktop) (5)
- ○ Mobile phone (2)
- ○ Tablet (3)
- ○ Other (please specify): (4) ____

End of Block: Mode of Completion

Start of Block: COVID-19 Screening Measure

Q66 How many separate COVID-19 infections have you had that were confirmed with a PCR or rapid COVID test?

- ○ 0 (1)
- ○ 1 (2)
- ○ 2 (3)
- ○ 3 (4)
- ○ 4 (5)
- ○ 5 or more (6)

---

*

Q47 Please specify the approximate date (mm/dd/yyyy) of your *first* COVID-19 infection.

____

Q4 How long was/is the duration of symptoms present?

▼ 0 - 4 weeks (4) ... Greater than 6 months (7)

Q70 Please specify the approximate date (mm/dd/yyyy) of your *most recent COVID-19* infection.

____

Q74 How long was/is the duration of symptoms present?

▼ 0 - 4 weeks (4) ... Greater than 6 months (7)

Q73 Are you still experiencing symptoms associated with a COVID-19 infection?

- ○ Yes (4)
- ○ No (5)
- ○ Not sure (6)

Q75 Which of the following symptoms are you still experiencing? (Please check all that apply)

- ☐ Abdominal pain (4)
- ☐ Alteration in smell (5)
- ☐ Alteration in taste (6)
- ☐ Anxiety (9)
- ☐ Panic attacks (31)
- ☐ Brain fog (10)
- ☐ Breathlessness and/or wheezing (11)
- ☐ Chest pain and/or chest tightness (12)
- ☐ Constipation (13)
- ☐ Cough (14)
- ☐ Depression (15)
- ☐ Diarrhea (16)
- ☐ Fatigue (17)
- ☐ Fevers (18)
- ☐ Hair loss (19)
- ☐ Headache (20)
- ☐ Heart palpitations (21)
- ☐ Joint pain (22)

- ☐ Loss of appetite (23)
- ☐ Muscle aches (24)
- ☐ Rash (25)
- ☐ Sleep disruptions or issues (26)
- ☐ Throat pain (27)
- ☐ Uncontrolled bladder (28)
- ☐ Other (please specify) (29) ____

Q76 How much have these symptoms negatively impacted your quality of life? (0 = not at all and 10 = very much so, affecting all or nearly all aspects of your life)

	Not at all	Very much so	Not Applicable
	0 1 2 3 4 5 6 7 8 9 10		
Yes (4)			

Q77 Have these symptoms impacted your employment situation or your ability to do your usual work?

- ○ Yes (4)
- ○ No (5)
- ○ Not applicable (6)

Q5 Were you ever prescribed any medications to treat COVID-19?

○ Yes (please specify what type below): (1) ____

○ No (2)

○ Not sure (3)

Q7 Did you require hospitalization?

○ Yes (please specify how long you were hospitalized): (1) ____

○ No (3)

Q61 Did you require treatment in the Intensive Care Unit (ICU)?

○ Yes (please specify for how long you were in the ICU): (1) ____

○ No (2)

Q8 Were you in need of mechanical ventilation?

○ Yes (please specify for how long): (1) ____

○ No, mechanical ventilation was not utilized (2)

Q9 Have you been diagnosed with any of the complications listed below?

	Yes (1)	No (2)
Long COVID (or long-hauler COVID) (19)	○	○
Unilateral or bilateral interstitial pneumonia (1)	○	○
Respiratory failure requiring mechanical ventilation (2)	○	○
Acute respiratory distress syndrome (ARDs) (3)	○	○
Heart inflammation (4)	○	○
Blood clots (5)	○	○
Thrombosis (16)	○	○
Septic shock (6)	○	○
Multiple organ dysfunction (MOD) or failure (MOF) (7)	○	○
Acute kidney injury (8)	○	○
Stroke (or brain hemorrhage) (9)	○	○
Encephalitis (10)	○	○
Guillain–Barré Syndrome (15)	○	○
Miller Fisher Syndrome (17)	○	○
Hypoxic ischemic injury (12)	○	○
Non-epileptic seizures (13)	○	○

Coma (14)	○	○

Page Break

COVID + Impact Do you believe that having COVID-19 impacted or continues to impact your personal life? If yes, how so? Here you may discuss any aspect of your personal life, for example, your health, well-being, housing, health insurance status, social support, etc.

____

____

____

____

____

COVID - Impact Do you believe that living amidst the COVID-19 pandemic impacted or continues to impact your personal life? If yes, how so? Here you may discuss any aspect of your personal life, for example, your health, well-being, housing, health insurance status, social support, etc.

____

____

____

____

____

Testing/Tx Barriers Have you experienced any barriers to COVID-19 testing or treatment? If so, what barriers did you experience and how have you managed dealing with them?

____

____

____

____

____

Page Break

Q62 The following are symptoms that people sometimes have after experiencing, witnessing, or being confronted with stressful or potentially traumatic events. Please read each statement carefully and indicate to what extent you have been bothered by the listed symptoms in the **past month** in relation to your experience with the COVID-19 pandemic. These experiences may have happened to you or to someone close to you (for example, family member, a friend, co-worker).

	Not at all (1)	Rarely (2)	Sometimes (3)	Most of the time (4)
Recurrent thoughts or memories of the COVID-19 pandemic (1)	o	o	o	o
Feelings as though the event is happening again (2)	o	o	o	o
Recurrent nightmares about having COVID-19 (3)	o	o	o	o
Sudden emotional or physical reactions when reminded of COVID-19 (4)	o	o	o	o
Avoiding activities that remind you of COVID-19 (5)	o	o	o	o
Avoiding thoughts or feelings associated with COVID-19 (6)	o	o	o	o
Feeling jumpy, easily startled (7)	o	o	o	o
Feeling on guard (8)	o	o	o	o

Q10 Please mark whether you have ever been diagnosed with any of the listed medical conditions.

	Yes (3)	No (4)
Asthma (1)	○	○
Obesity or severe obesity (2)	○	○
High blood pressure (3)	○	○
Heart disease (coronary artery disease, cardiomyopathy, heart failure) (4)	○	○
Chronic obstructive pulmonary disease (COPD) (5)	○	○
Type 1 or 2 diabetes (6)	○	○
Liver disease (7)	○	○
Cancer (8)	○	○
Brain and/or nervous system conditions (9)	○	○
Post-traumatic stress disorder (10)	○	○
Other (Please fill in) (11)	○	○

Page Break

Q11 Under each heading, please mark one box that best describes your health today.

Mobility

- ◯ I have no problems in walking about (1)
- ◯ I have slight problems in walking about (2)
- ◯ I have moderate problems in walking about (3)
- ◯ I have severe problems in walking about (4)
- ◯ I am unable to walk about (5)

Q12 Self-care

- ◯ I have no problems washing or dressing myself (1)
- ◯ I have slight problems washing or dressing myself (2)
- ◯ I have moderate problems washing or dressing myself (3)
- ◯ I have severe problems washing or dressing myself (4)
- ◯ I am unable to wash or dress myself (5)

Q13 Usual activities (e.g., work, study, housework, family or leisure activities)

- ◯ I have no problems doing my usual activities (1)
- ◯ I have slight problems doing my usual activities (2)
- ◯ I have moderate problems doing my usual activities (3)
- ◯ I have severe problems doing my usual activities (4)
- ◯ I am unable to do my usual activities (5)

Q14 Pain/Discomfort

- ○ I have no pain or discomfort (1)
- ○ I have slight pain or discomfort (2)
- ○ I have moderate pain or discomfort (3)
- ○ I have severe pain or discomfort (4)
- ○ I have extreme pain or discomfort (5)

Q15 Anxiety/Depression

- ○ I am not anxious or depressed (1)
- ○ I am slightly anxious or depressed (2)
- ○ I am moderately anxious or depressed (3)
- ○ I am severely anxious or depressed (4)
- ○ I am extremely anxious or depressed (5)

Q17 We would like to know how good or bad your health is today. This scale is numbered from 0-100, with 0 meaning the worst health you can imagine, and 100 meaning the best health you can imagine. Please move the slider below the scale to indicate how your health is today.

	Extremely bad	Somewhat bad	Neither good nor bad	Somewhat good	Extremely good
	0 10 20 30 40 50 60 70 80 90 100				
Your health today (1)					

Q63 We would like to know how old you feel. Please use this drop down menu to select the age in years that you feel.

▼ 0 (1) ... 120 (123)

Page Break

Q41 Have you experienced a loss or change in taste or smell (with an onset in the last 24 months)?

○ Yes (1)

○ No (2)

Page Break

Q42 Which best describes your loss of smell?

- ◯ I was not able to smell anything at all (even intense scents such as bleach) (1)
- ◯ I experienced a partial loss of smell (2)
- ◯ I did not experience any loss of smell (3)

Q44 Which best describes your loss of taste?

- ◯ I was not able to taste anything at all (even to the point of not knowing what I had in my mouth if I closed my eyes) (1)
- ◯ I experienced a partial loss of taste (2)
- ◯ I did not experience any loss of taste (3)

Q45 Maybe you were capable of smelling, but you perceived some known scents as strange, even to the point of confusing them with your eyes closed. Did this happen to you?

- ◯ Yes (1)
- ◯ No (2)

Q46 Maybe you were capable of tasting foods, but you perceived some known flavors as strange (even to the point of confusing them with your eyes closed). Did this happen to you?

- ◯ Yes (1)
- ◯ No (2)

Q47 Are you CURRENTLY experiencing a loss or change in taste or smell? If yes, please specify how long you have been experiencing a loss or change. If no, please specify how long the loss or change lasted.

- Yes (1) ____

- No (2) ____

End of Block: COVID-19 Screening Measure

Start of Block: PSS-12

Q18 The questions on this scale ask you about your feelings and thoughts during the COVID-19 pandemic. Please indicate how often you felt or thought a certain way.

	Never (1)	Almost never (2)	Sometimes (3)	Fairly often (4)	Very often (5)
Felt upset about the unexpected changes resulting from COVID-19 (1)	○	○	○	○	○
Felt that you were unable to control the important things in your life (2)	○	○	○	○	○
Felt nervous and stressed (3)	○	○	○	○	○
Felt overwhelmed and/or panicked (4)	○	○	○	○	○
Felt like it is difficult to relax (5)	○	○	○	○	○
Felt confident about your ability to handle your personal life (6)	○	○	○	○	○
For psychometric purposes, please select "Fairly often" for this item. (7)	○	○	○	○	○
Felt that things were going your way (8)	○	○	○	○	○

Found that you could not cope with all the things that you had to do (9)	○	○	○	○	○
Been able to control irritations in your life (10)	○	○	○	○	○
Felt that you were on top of things (11)	○	○	○	○	○
Been angered because of things outside of your control (12)	○	○	○	○	○
Felt difficulties were piling up so high that you could not overcome them (13)	○	○	○	○	○
Felt scared learning about the pandemic on the news, social media, from your school or place of work or through other interactions (14)	○	○	○	○	○

Q19 The COVID-19 pandemic is/has been disruptive to your life.

- ◯ Disagree (1)
- ◯ Slightly disagree (2)
- ◯ Neither agree or disagree (3)
- ◯ Slightly agree (4)
- ◯ Agree (5)

End of Block: PSS-12

Start of Block: Subjective Cognition

Q38 Has your general cognitive functioning (for example, ability to concentrate, experience of brain fog, etc.) changed during the COVID-19 pandemic?

- ◯ Yes, it has decreased (1)
- ◯ No, it has not changed (2)
- ◯ Yes, it has improved (3)

Q39 Compared to before the start of the COVID-19 pandemic, how often have you experienced the following?

	A lot less than before (1)	Less than before (2)	Not more or less than before (3)	More than before (4)	A lot more than before (5)
Difficulty remembering things (1)	○	○	○	○	○
Difficulty concentrating on something (2)	○	○	○	○	○
Difficulty doing two things at the same time (3)	○	○	○	○	○
Difficulty recalling things (4)	○	○	○	○	○
Forgetfulness (5)	○	○	○	○	○

Q20 Compared to before the start of the COVID-19 pandemic, how much have you been bothered by each of the following?

	Not at all (1)	A little (2)	Somewhat (3)	Quite a lot (4)	Very much (5)
Spells of confusion (1)	○	○	○	○	○
Thoughts getting mixed up (2)	○	○	○	○	○
Poor concentration (3)	○	○	○	○	○
Can't easily make decisions (4)	○	○	○	○	○
Poor memory for recent events (5)	○	○	○	○	○
For psychometric purposes, please select "A little" for this item. (6)	○	○	○	○	○
Can't take things in when speaking to people (7)	○	○	○	○	○
Thoughts are slow (8)	○	○	○	○	○
Muzzy head (9)	○	○	○	○	○
Can't find right words (10)	○	○	○	○	○

End of Block: Subjective Cognition

Start of Block: Mood and Sleep Self-Reports

Q58 Over the **last 2 weeks**, how often have you been bothered by the following problems?

	Not at all (1)	Several days (2)	More than half the days (3)	Nearly every day (4)
Feeling nervous, anxious or on edge (1)	○	○	○	○
Not being able to stop or control worrying (2)	○	○	○	○
Little interest or pleasure in doing things (3)	○	○	○	○
Feeling down, depressed or hopeless (4)	○	○	○	○

Page Break

Q60 The following questions relate to your usual sleep habits during the past month only. Your answers should indicate the most accurate reply for the majority of days and nights in the past month. Please answer all questions.

Q61 During the past month, when have you usually gone to bed at night? (please specify a.m. or p.m. in your response)

____

Q62 During the past month, when have you usually gotten up in the morning? (please specify a.m. or p.m. in your response)

____

Q63 During the past month, how long has it usually taken you (in minutes) to fall asleep each night?

____

Q64 During the past month, how many hours of actual sleep did you get at night? (This may be different than the number of hours you spend in bed).

____

Q65 During the past month, how often have you had trouble sleeping because you wake up in the middle of the night or early morning?

- ◯ Not during the past month (1)
- ◯ Less than once a week (2)
- ◯ Once or twice a week (3)
- ◯ Three or more times a week (4)

Q66 During the past month, how would you rate your sleep quality overall?

- ◯ Very good (1)
- ◯ Fairly good (4)
- ◯ Fairly bad (5)
- ◯ Very bad (6)

Q68 Please indicate your agreement with each of the following items.

	Strongly disagree (1)	Disagree (2)	Slightly disagree (3)	Neither agree or disagree (4)	Slightly agree (5)	Agree (6)	Strongly agree (7)
In most ways my life is close to my ideal. (1)	○	○	○	○	○	○	○
The conditions of my life are excellent. (2)	○	○	○	○	○	○	○
I am satisfied with my life. (3)	○	○	○	○	○	○	○
For psychometric purposes, please select "Disagree" for this item. (4)	○	○	○	○	○	○	○
So far I have gotten the important things I want in life. (5)	○	○	○	○	○	○	○
If I could live my life over, I would change almost nothing. (6)	○	○	○	○	○	○	○

Q69 Thinking about yourself and how you normally feel, to what extent do you generally feel:

	Never (1) (1)	(2) (2)	(3) (3)	(4) (4)	Always (5) (5)
Upset (1)	○	○	○	○	○
Hostile (2)	○	○	○	○	○
Alert (3)	○	○	○	○	○
Ashamed (4)	○	○	○	○	○
Inspired (5)	○	○	○	○	○
Nervous (6)	○	○	○	○	○
Determined (7)	○	○	○	○	○
Attentive (8)	○	○	○	○	○
Afraid (9)	○	○	○	○	○
Active (10)	○	○	○	○	○

Hopefulness People vary in how hopeful they feel about the future. Please indicate how much you agree with the following statement.

	Strongly disagree (1)	Disagree (2)	Neither agree nor disagree (3)	Agree (4)	Strongly agree (5)
I am hopeful about the future following COVID-19 (1)	o	o	o	o	o

End of Block: Mood and Sleep Self-Reports

Start of Block: Demographics

Q21 How old did you turn on your last birthday?

▼ 18 (85) ... 120 (187)

Q64 Have you ever experienced a concussion?

- Yes (if yes, how many concussions have you experienced?) (1) ____
- No (2)
- Not sure (3)

Q65 Please provide the approximate date(s) of your concussion(s) here. (Leave this text box empty if you have not experienced a concussion.)

____

Q22 What is your race or ethnic origin? Select all that apply.

- ☐ White (1)
- ☐ Black or African American (2)
- ☐ Hispanic, Latino or Spanish origin (3)
- ☐ American Indian or Alaskan Native (4)
- ☐ Asian or Asian American and/or Pacific Islander (5)
- ☐ Native Hawaiian or Pacific Islander (6)
- ☐ Other (please specify) (7) ____

Q23 What is your gender?

- ○ Male (1)
- ○ Female (2)
- ○ Trans male/Trans man (3)
- ○ Trans female/Trans woman (4)
- ○ Genderqueer/Gender non-conforming (5)
- ○ Non-binary (6)
- ○ Different identity (please state below) (7) ____

Q25 How many years of education have you completed (for example, completing high school = 12 years; completing a 4-year college degree = 16 years)

____

Q48 What is your current employment status?

- ☐ Employed full-time (40 or more hours per week) (1)
- ☐ Employed part-time (up to 39 hours per week) (2)
- ☐ Unemployed and currently looking for work (3)
- ☐ Unemployed and not currently looking for work (4)
- ☐ Student (5)
- ☐ Retired (6)
- ☐ Homemaker (7)
- ☐ Self-employed (8)
- ☐ Unable to work (9)
- ☐ Other (please specify) (10) ____

Q49 Has the COVID-19 pandemic affected your employment status?

- ○ No (1)
- ○ Yes (2)
- ○ Not applicable (3)

Q50 Are you considered an essential employee in the state in which you are employed?

- ○ No (1)
- ○ Yes (2)
- ○ Not applicable (3)

Q52 Do you work in a healthcare setting?

- ○ No (1)
- ○ Yes (2)
- ○ Not applicable (3)

Q54 How much total combined money did all members of your household earn in 2020?

- ◯ $0 - $9,999 (1)
- ◯ $10,000- $19,999 (2)
- ◯ $20,000- $29,999 (3)
- ◯ $30,000- $39,999 (4)
- ◯ $40,000 - $49,999 (5)
- ◯ $50,000- $59,999 (6)
- ◯ $60,000- $69,999 (7)
- ◯ $70,000- $79,999 (8)
- ◯ $80,000- $89,999 (9)
- ◯ $90,000- $99,999 (10)
- ◯ $100,000 or more (11)

Q35 Which state do you currently live in?

▼ Alabama (1) ... Wyoming (50)

Q34 In what language do you speak most often?

____

Q63 Where did you hear about this study?

○ ResearchMatch.org (1)

○ Social media (2)

○ Other (please specify) (4) ____

Q56 Have you been vaccinated against COVID-19?

○ Yes (1)

○ No (2)

Q57 What month and year did you receive your initial vaccination? If you received multiple doses (e.g., Pfizer vaccinations included two doses), please specify the date of your last dose, and the number of doses.

____

Q67 Have you received a COVID-19 booster vaccination?

○ Yes (please specify how many booster vaccinations you have received) (1) ____

○ No (2)

○ Not sure (3)

Further Experiences Is there anything else you would like to share in relation to the COVID-19 pandemic that was not captured in this study?

____

Page Break

Q37 Are you willing to be contacted at a later time to complete additional surveys and/or cognitive tasks? (By answering yes now you are under no obligation to complete any study measures in the future)

- ☐ Yes (1)
- ☐ No (2)

Q38 Please provide your email address here.

____

End of Block: Demographics
